# Transcriptome and metabolite analyses in *Azadirachta indica:* identification of genes involved in biosynthesis of bioactive triterpenoids

**DOI:** 10.1038/s41598-017-05291-3

**Published:** 2017-07-11

**Authors:** Sweta Bhambhani, Deepika Lakhwani, Parul Gupta, Ashutosh Pandey, Yogeshwar Vikram Dhar, Sumit Kumar Bag, Mehar Hasan Asif, Prabodh Kumar Trivedi

**Affiliations:** 1 CSIR-National Botanical Research Institute, Council of Scientific and Industrial Research (CSIR-NBRI), Rana Pratap Marg, Lucknow, 226 001 India; 2 Academy of Scientific and Innovative Research (AcSIR), Anusandhan Bhawan, 2 Rafi Marg, New Delhi, 110 001 India; 3 Botany & Plant Pathology, Oregon State University, Corvallis, 97331 USA; 4 Faculty of Biology, University of Bielefeld, Universitätsstraße 25, 33615 Bielefeld, Germany

## Abstract

*Azadirachta indica* A. Juss, commonly known as Neem, is the reservoir of triterpenoids of economic importance. Metabolite analysis of different developmental stages of leaf and fruit suggests tissue-specific accumulation of the major triterpenoids in this important tree. Though biosynthesis of these complex molecules requires substrate flux from the isoprenoid pathway, enzymes involved in late biosynthetic steps remain uncharacterized. We established and analyzed transcriptome datasets from leaf and fruit and identified members of gene families involved in intermediate steps of terpenoid backbone biosynthesis and those related to secondary transformation leading to the tissue-specific triterpenoid biosynthesis. Expression analysis suggests differential expression of number of genes between leaf and fruit and probable participation in the biosynthesis of fruit-specific triterpenoids. Genome-wide analysis also identified members of gene families putatively involved in secondary modifications in late biosynthetic steps leading to the synthesis of highly oxygenated triterpenoids. Expression and molecular docking analyses suggest involvement of specific members of CYP450 family in secondary modifications for the biosynthesis of bioactive triterpenoids. This study generated rich genomic resource and identified genes involved in biosynthesis of important molecules, which will aid in the advancement of tools for functional genomics and elucidation of the biosynthesis of triterpenoid from this important tree.

## Introduction

Neem (*Azadirachta indica* A. Juss) belonging to family Meliaceae is the richest source of bioactive molecules specially tetranortriterpenoids (limonoids). More than 150 tetranortriterpenoids with diverse skeletal structures isolated from different parts of this tree have been grouped under ring-intact and C-seco triterpenoids^1–3^. The C-seco triterpenoids include azadirachtin, nimbin and salannin^4^, which make this tree useful for the healthcare and agronomical purposes. Studies also suggest that the abundance of these structurally distinct tetranortriterpenoids is highly tissue-specific^3^. Studies suggest use of Neem extract for the treatment of variety of diseases including malaria, rheumatism, gastric ulcer, cardiovascular diseases, osteoporosis and vaginal infections as well as mosquito repellent^5–8^. In addition, tetranortriterpenoids from Neem serve as agrochemicals due to their active role as insect feeding deterrants, toxicants, growth-disruptants as well as hamper the development of large range of insects, pests and nematodes^9^. Although the putative biosynthetic pathway for the formation of Neem tetranortriterpenoids has been predicted^3, 10^, very limited information is available about intermediate steps, enzymes and regulation of this important pathway.

Tetranortriterpenes are synthesized *via* isoprenoid biosynthesis pathway with basic building blocks such as isopentenyl diphosphate (IPP) and dimethylallyl diphosphate (DMAPP), which is synthesized through the mevalonate (MVA)/methylerythritol phosphate (MEP) or both the pathways^11^. Studies suggest tirucallol as one of the precursor molecule for biosynthesis of tetranortriterpenes, which is formed from two units of farnesyl diphosphate (FPP) and subsequently leads to the synthesis of butyrospermol by allylic isomerization^12^. The butyrospermol gets oxidized and subsequently undergoes Wagner-Meerwein 1,2-methyl shift rearrangement to form apotirucallol^13, 14^ which leads to the synthesis of tetranortriterpenoids by cleavage of four terminal carbons from the side chain^14^. Out of different tetranortriterpenoids, biosynthesis of major bioactive molecule azadirachtin involves several uncharacterized steps involving oxidation, cyclization and epoxidation reactions.

Owing to the commercial importance of tetranortriterpenoids, few studies have been carried out to generate the genomic resource of Neem tree^3, 4, 15–18^. These studies have been helpful in identifying few genes encoding enzymes involved in early biosynthetic pathways (MVA and MEP) leading to the formation of precursor triterpenoids. However, comprehensive analysis related to genes and enzymes involved in late biosynthetic pathway has not been carried out. It is presumed that the correlation of metabolic profiles with expression of genes from different tissues may help in identification of members of gene families involved in late steps of triterpenoid biosynthesis in Neem. In this study, to explore the biosynthesis pathway of tetranortriterpenoids, transcriptome datasets of leaf and fruit tissues synthesizing distinct molecules were established and analyzed. Using transcriptome datasets and metabolic profiling, genes encoding enzymes involved in MVA and MEP pathways and members of Cytochrome P450 (CYP) family, which could be putatively involved in tetranortriterpenoid biosynthesis, have been identified. Based on the expression analysis, tissue-specific azadirachtin accumulation and molecular docking approaches, involvement of a set of specific CYP450s has been proposed in the biosynthesis of tetranortriterpenoids in Neem.

## Results

### Phytochemical analysis of tetranortriterpenoids in various plant parts

**Figure 1:**
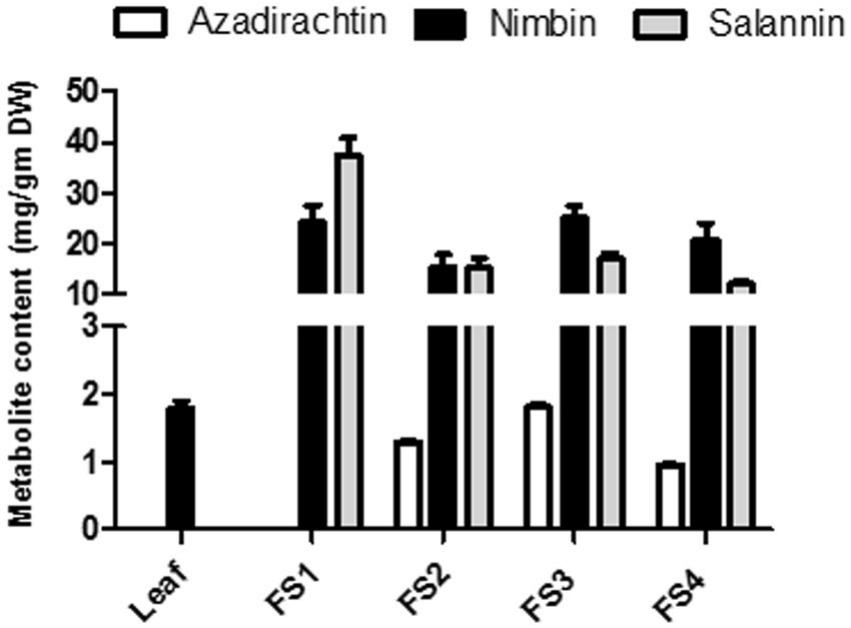
Phytochemical analysis of different plant parts of *Azadirachta indica*. Leaf, Mature leaf; FS1, Fruit stage 1; FS2, Fruit stage-2; FS3, Fruit stage-3 and FS4, Fruit stage-4.

Phytochemical analysis of tetranortriterpenoids in leaf and various developmental stages of fruit (Fig. 1) suggested fruit-specific accumulation of azadirachtin with highest content in FS3 stage. Though nimbin was detectable in leaf however its accumulation was several folds higher in different stages of fruit. Azadirachtin and salannin were not detected in leaf tissue which also contained minimum amount of nimbin. This suggests that fruit (FS3) contains maximum whereas leaf is the poorest source of these important phytochemicals.

### Establishment of transcriptome datasets, assembly and annotation

cDNA libraries prepared using total RNA from mature leaf (ML) and fruit (FS3) were sequenced on a 454-GS FLX Titanium system. Transcriptome sequencing generated 8,49,781 (273.8 Mb) and 7,07,392 (220 Mb) high quality reads for leaf and fruit tissues respectively (Supplementary Table S1). The average read length for the ML and FS3 was 322.2 bp and 310.9 bp respectively. From ML library, 6,97,725 reads assembled into 18,172 contigs and 48,163 remained as singletons. Similarly, 5,63,212 reads for FS3 assembled into 14,566 contigs and 46,310 remained as singletons. Average length of contig in ML and FS3 was 732.4 bp and 687.1 bp respectively. Among all the contigs, 65.9% from ML and 64.4% from FS3 were considered as large contigs. Average GC content for contigs in both the libraries was 41%. Total transcripts for ML and FS3 libraries were 66,335 and 60,876 respectively. The total number of unigenes annotated by AGIProt is 34,898 and 30,385 for ML and FS3 respectively. Similarly, 38,977 and 33,960 unigenes were annotated by NR for ML and FS3 respectively. The total number of gene families annotated by Pfam is 23,265 and 20,110 for ML and FS3, respectively (Supplementary Table S2).

### Gene ontology and functional classification

**Figure 2:**
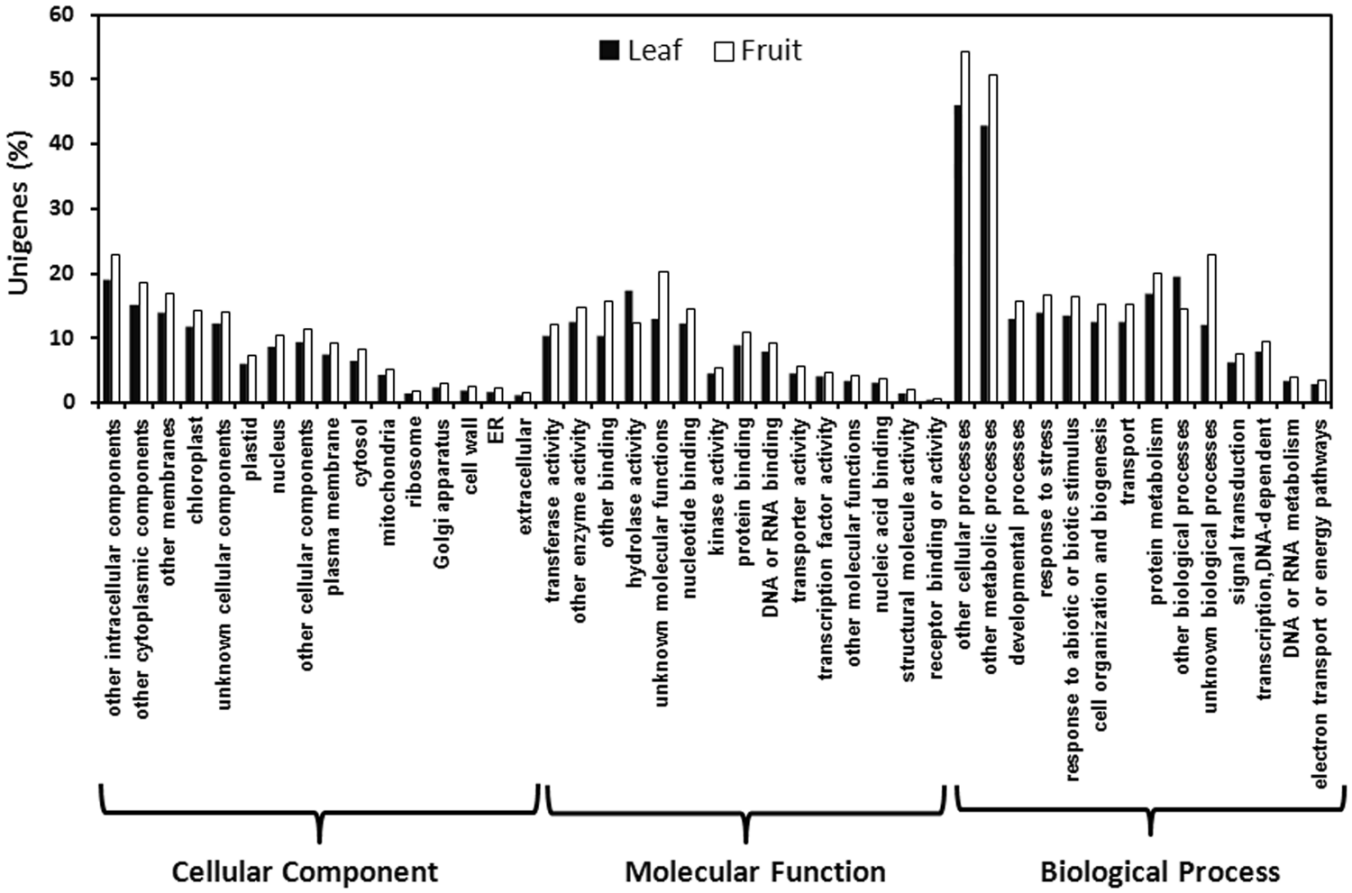
Histogram representing gene ontology classification. Unigenes have been grouped under three main categories: biological process, cellular component and molecular function. Black and white bars represent the percent number of assignments of unigenes present in leaf and fruit respectively. BLAST matches in the TAIR10 database was carried out for each GO term.

The function of unigenes present in ML and FS3 libraries was classified by GO assignments. Based on sequence homology, 13,861 and 13,106 sequences (among the unigenes annotated by AGI prot) from ML and FS3 libraries respectively were categorized into 45 functional groups under different categories of biological process, cellular component and molecular function (Fig. 2). Among which, genes in terms of ‘other cellular processes’, ‘other metabolic processes’ and ‘other intracellular components’ categories were higher in number. Lower number of genes was attributed to groups of ‘receptor binding or activity’, ‘ER’, ‘Golgi apparatus’ and ‘Extracellular’ category. The pattern of distribution of unigenes showed significant difference under various GO terms in ML and FS3. As higher number of unigenes was designated to ‘other metabolic processes’ in ML and FS3 libraries, these datasets can be helpful in identifying novel genes participating in the biosynthesis of triterpenoid in *A*. *indica*.

Kyoto Encyclopedia of Genes and Genomes (KEGG) was used to perform functional classification and pathway assignment. Interestingly, 1,437 and 1,541 unigenes from FS3 and ML libraries were identified to be involved in various secondary metabolite pathways (Table 1). The cluster of ‘limonene and pinene degradation’ (404 members) amongst all secondary plant product biosynthesis pathways formed the largest group followed by phenylpropanoid biosynthesis (289 members) in case of FS3. Contrastingly in ML library, the cluster of ‘phenylpropanoid biosynthesis’ (352 members) forms the largest group followed by ‘limonene and pinene degradation’ (283 members). ‘Terpenoid backbone biosynthesis’ group included 80 and 87 members for FS3 and ML libraries respectively.

**Table 1:** Unigenes associated with biosynthesis of secondary metabolites in fruit and leaf.

Biosynthesis of Other Secondary Metabolites	Fruit	Leaf
Anthocyanin Biosynthesis	27	32
Brassinosteroid Biosynthesis	23	27
Caffeine Metabolism	2	2
Carotenoid Biosynthesis	62	62
Diterpenoid Biosynthesis	26	33
Flavonoid Biosynthesis	95	107
Flavone and Flavonol Biosynthesis	22	37
Glucosinolate Biosynthesis	35	35
Indole Alkaloid Biosynthesis	17	22
Isoquinoline Alkaloid Biosynthesis	156	203
Monoterpenoid biosynthesis	5	11
Limonene and pinene degradation	404	283
Novabiocin Biosynthesis	136	168
Phenylpropanoid Biosynthesis	289	352
Sesquiterpenoid and Triterpenoid Biosynthesis	8	13
Terpenoid backbone Biosynthesis	80	87
Tetracycline Biosynthesis	36	36
Zeatin Biosynthesis	14	31
Total	1437	1541

### Identification of genes related to terpenoid backbone biosynthesis

**Figure 3:**
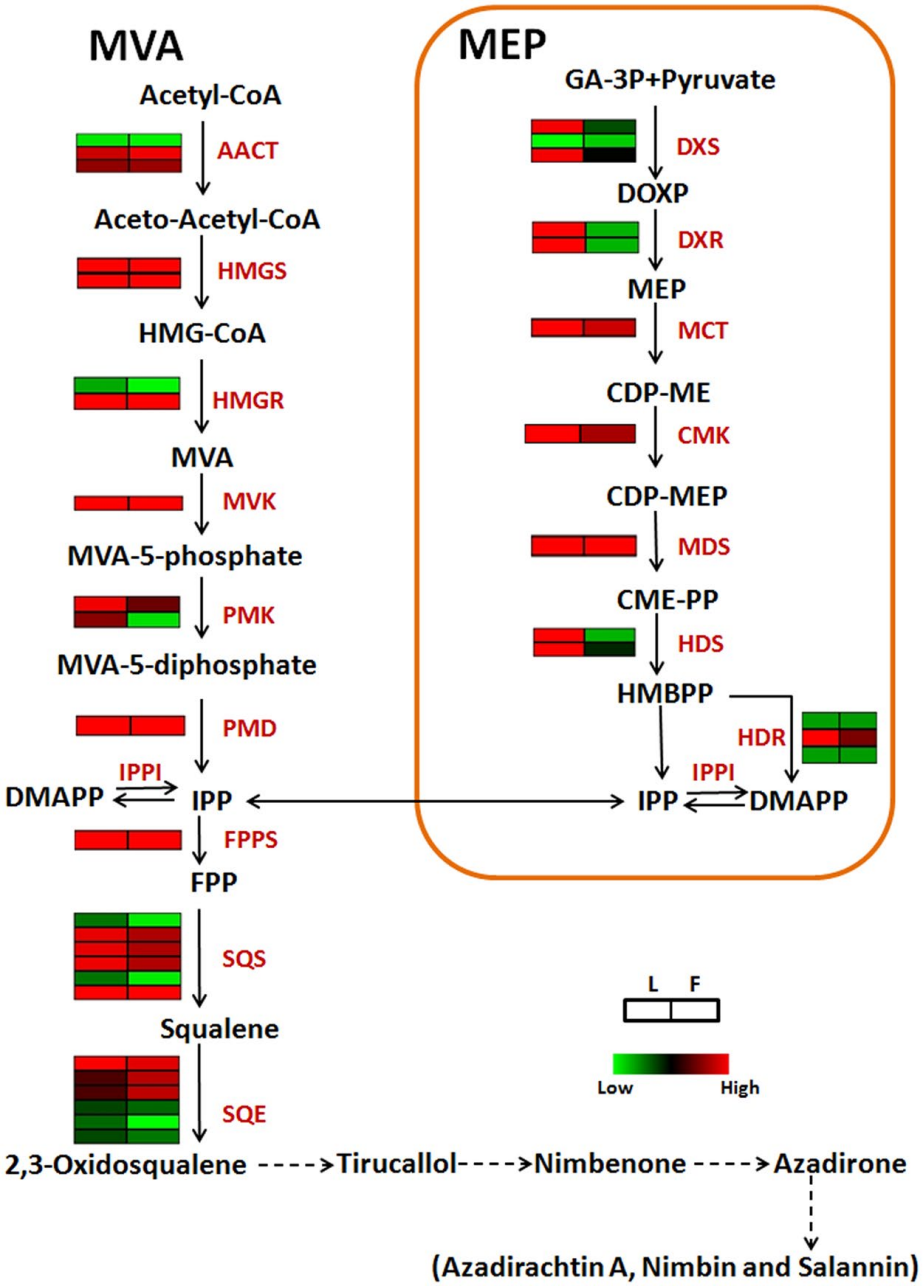
Putative pathway for tetranortriterpenoid biosynthesis from *Azadirachta indica*. Name of the enzymes catalyzing biochemical steps are shown between the reactions and expression of unigenes encoding these enzymes in leaf and fruit is shown by heatmap. Broken arrows represent putative steps involving enzymes involved in secondary modifications. AACT, acetyl CoA acetyltransferase; HMGS, 3-hydroxy-3-methylglutaryl-coenzymeA synthase; HMGR, 3-hydroxy-3-methylglutaryl-coenzymeA reductase; MVK, mevalonate kinase; PMK, phosphomevalonate kinase; PMD, phosphomevalonate decarboxylase; DXS, 1-deoxy-D-xylulose-5-phosphate synthase; DXR, 1-deoxy-D-xylulose-5-phosphate reductoisomerase; MCT, 2-C-methyl-D-erythritol-4-phosphate cytidylyl transferase; CMK, 4-diphosphocytidyl-2-C-methyl-D-erythritol kinase; MDS, 2-C-methyl-D-erythritol-2,4-cyclodiphosphate synthase; HDS, 4-hydroxy-3-methylbut-2-enyldiphosphate synthase; HDR, 4-hydroxy-3-methylbut-2-enyldiphosphate reductase; IPPI, isopentenyl diphosphate isomerase; FPPS, farnesyl diphosphate synthase; SQS, squalene synthase; SQE, squalene monooxygenase/epoxidase.

Terpenoids are synthesized by isoprenoid biosynthesis pathway, which generates a universal building block IPP and its isomer DMAPP for all the terpenes. This universal precursor is produced ‘via’ MVA and MEP pathways. In total, generation of IPP involves 14 intermediate steps (MVA and MEP). To identify transcripts related to all the intermediate genes and differential gene expression analysis, a combined assembly using ML and FS3 datasets was carried out. Using this combined assembly and annotation through different databases, all the genes encoding enzymes involved in these reactions were identified and most of the enzymes were encoded by more than one unigenes (Table 2). Maximum number of unigenes (six) was identified for Squalene synthase (SQS) and Squalene monoxygenase/epoxidase (SQE). Expression pattern of each gene in both the libraries was analyzed by contigs acquired from combined assembly, due to the presence of these genes in both the libraries. Analysis revealed that majority of genes participating in triterpenoid backbone biosynthesis express differentially in ML and FS3 (Fig. 3).

**Table 2:** Details of enzymes involved in triterpenoid biosynthesis.

Enzyme	EC	Abbreviation	No. of unigenes
Acetyl CoA acetyltransferase	2.3.1.9	AACT	3
3-hydroxy-3-methylglutaryl-coenzymeA synthase	2.3.3.10	HMGS	2
3-hydroxy-3-methylglutaryl-coenzymeA reductase	1.1.1.34	HMGR	2
Mevalonate kinase	2.7.1.36	MVK	1
Phosphomevalonate kinase	2.7.4.2	PMK	2
Phosphomevalonate decarboxylase	4.1.1.33	PMD	1
1-deoxy-D-xylulose-5-phosphate synthase	2.2.1.7	DXS	3
1-deoxy-D-xylulose-5-phosphate reductoisomerase	1.1.1.267	DXR	2
2-C-methyl-D-erythritol-4-phosphate cytidylyl transferase	2.7.7.60	MCT	1
4-diphosphocytidyl-2-C-methyl-D-erythritol kinase	2.7.1.148	CMK	1
2-C-methyl-D-erythritol-2,4-cyclodiphosphate synthase	4.6.1.12	MDS	1
4-hydroxy-3-methylbut-2-enyldiphosphate synthase	1.17.7.1	HDS	2
4-hydroxy-3-methylbut-2-enyldiphosphate reductase	1.17.1.2	HDR	3
Farnesyl diphosphate synthase	2.5.1.10	FPPS	1
Squalene synthase	2.5.1.21	SQS	6
Squalene monooxygenase/epoxidase	1.14.99.7	SQE	6

### Expression analysis of genes of MVA and MEP pathways in different plant parts by qRT-PCR

**Figure 4:**
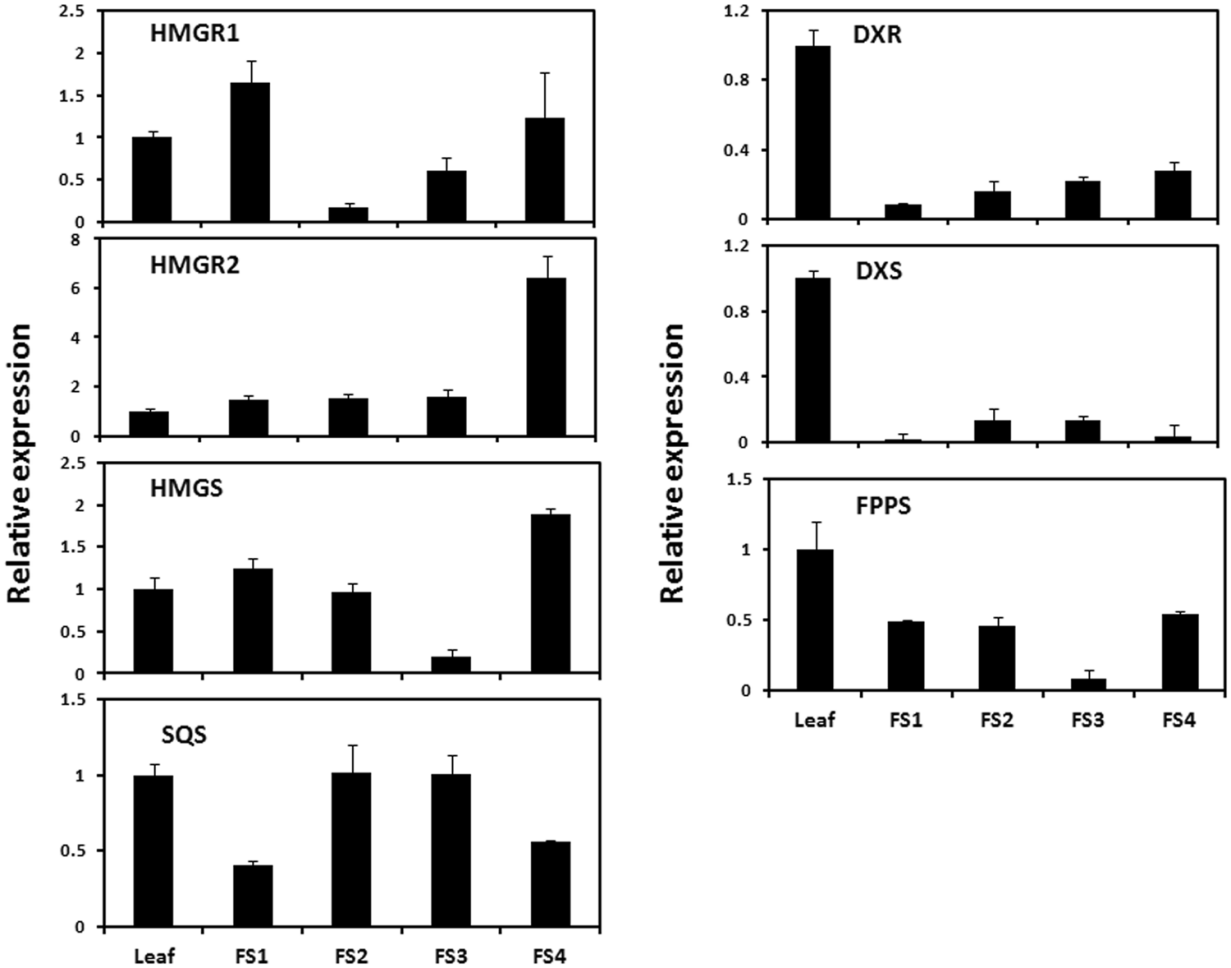
Expression analysis of genes involved in MVA and MEP pathways. Expression was analyzed through qRT-PCR in different plant parts. Leaf, Mature leaf; FS1, Fruit stage 1; FS2, Fruit stage-2; FS3, Fruit stage-3 and FS4, Fruit stage-4. *HMGR1* and *HMGR2*, *3-Hydroxy-3-methylglutaryl-coenzymeA reductase*; *HMGS*, *3-Hydroxy-3-methylglutaryl-coenzymeA synthase*; *DXR*, *1-Deoxy-D-xylulose-5-phosphate reductoisomerase*; *DXS*, *1-Deoxy-D-xylulose-5-phosphate synthase*; *FPPS*, *Farnesyl diphosphate synthase*; *SQS*, *Squalene synthase*.

The expression of various genes of MVA and MEP pathways (*HMGR1*, *HMGR2*, *HMGS*, *DXR*, *DXS*, *SQS* and *FPPS*) was analyzed in leaf and fruit (FS1, FS2, FS3, FS4) tissues of *A*. *indica* through quantitative real time PCR (Fig. 4). Genes of MVA and MEP pathways expressed differentially in developmental stages of leaf and fruit. Significantly lower expression of genes involved in MEP pathway (*AiDXS* and *AiDXR*) was observed in all stages of fruit as compared to leaf. Though genes of MVA pathway (*AiHMGR2* and *AiHMGS*) showed higher transcript level in FS4 as compared to leaf, expression of *AiHMGR1* was highest in FS1. The expression of *AiFPPS* was highest in leaf and decreased in all the stages of fruit. The expression of *AiSQS* was higher in leaf comparable to fruit (FS2 and FS3). These results also validated differential expression pattern of genes as observed from the transcriptome data analysis.

### Genes associated with secondary modifications and biosynthesis of tetranortriterpenoids

**Figure 5:**
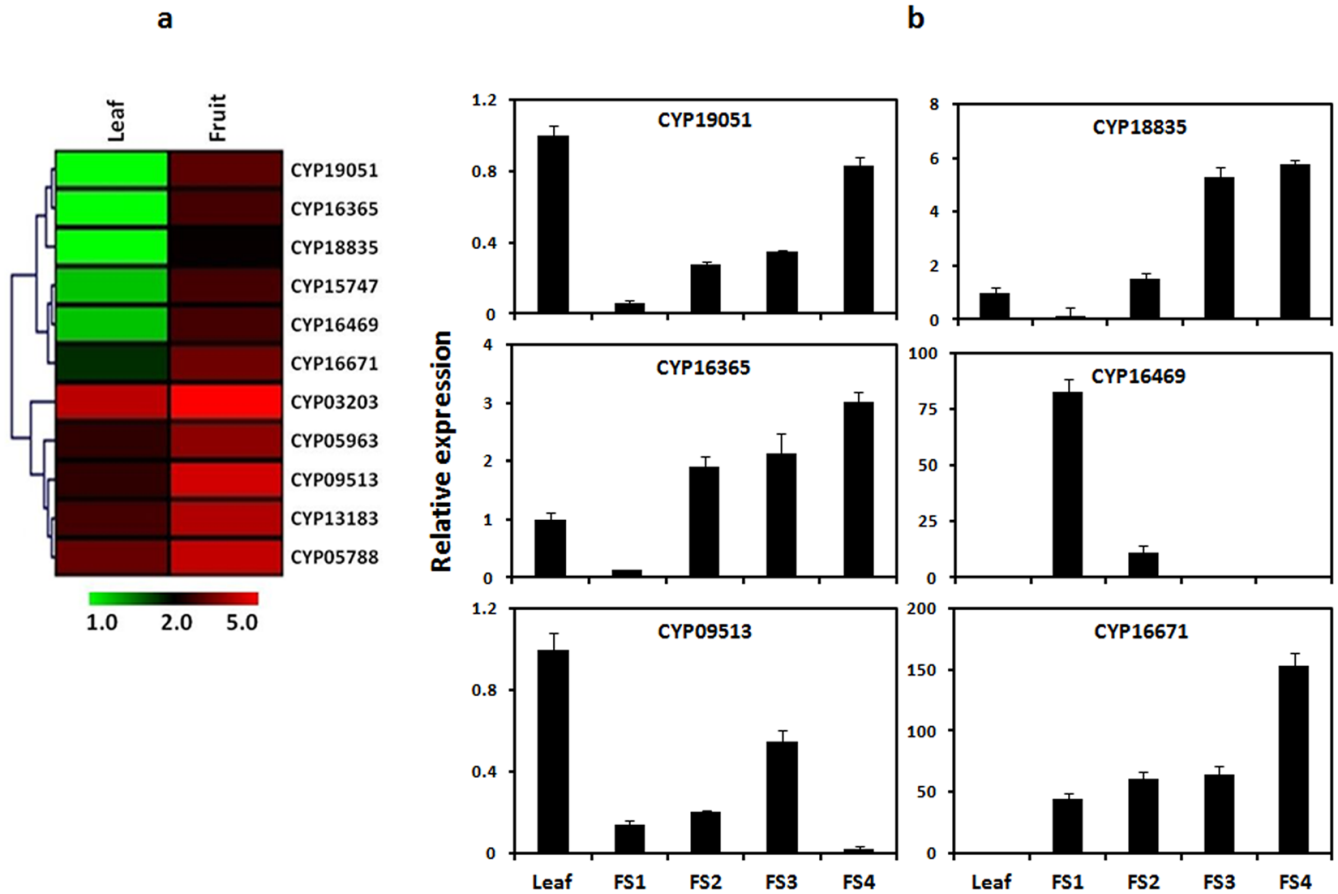
Identification and differential gene expression of members of CYP450 gene family putatively involved in triterpenoid biosynthesis. (**a**) Differentially expressed members of CYP450 gene family. Two columns represent leaf and fruit. Individual contigs encoding different members of CYP450 gene family are represented in rows. For each contig, log_2_tpm value obtained in leaf and fruit transcriptome datasets was used for clustering to visualize differential expression. (**b**) Validation of differential expression of selected members of CYP450 gene family by qRT-PCR in different plant parts. Leaf, Mature leaf; FS1, Fruit stage 1; FS2, Fruit stage-2; FS3, Fruit stage-3 and FS4, Fruit stage-4.

Phytochemical analysis suggested that azadirachtin specifically accumulates in fruit (FS3) and not in the leaf tissue (Fig. 1). This differential synthesis of azadirachtin might be due to expression of genes encoding enzymes putatively involved in catalyzing reactions in the biosynthesis of triterpenoids and secondary transformation include cytochrome P450s (CYPs), glycosyltransferases (GTs) and methyltransferases (MTs)^19^. As molecules present in Neem are highly oxygenated triterpenoids, identification of differentially expressed CYP450s may suggest their involvement in fruit-specific synthesis of azadirachtin and other triterpenoids. Through annotation using different databases, in leaf and fruit libraries, the numbers of contigs encoding CYP450s were 170 and 149 respectively (Supplementary Table S3). As putative azadirachtin biosynthesis may involve intermediate steps catalyzed by CYP450s, expression was analyzed in leaf and fruit tissues for the identified members of the CYP450 family (Fig. 5a). Differential expression pattern for these genes in different tissues may be one of the reasons for the differential accumulation of specific triterpenoids in leaf and fruit tissues.

Analysis led to the identification of a few members of CYP450 gene family which are highly expressed in fruit as compared to leaf. These might be involved in intermediate steps for the biosynthesis of tetranortriterpenoid in Neem. Further, to validate their differential expression, six CYP450s were selected (based on significantly higher abundance in FS3 as compared to leaf datasets) (Fig. 5a and b). The expression of these selected members of CYP gene family varied in developmental stages of plant tissues. The expression of *CYP16365*, *CYP16671* and *CYP18835* consistently increased in different fruit stages (FS1 to FS4) as compared to leaf. *CYP19051* was highly expressed in leaf, although there was increase in its expression in developmental stages of fruit. The expression of *CYP16469* was the maximum in fruit FS1 and that of *CYP09513* was maximum in leaf. This study suggested that the expression of *CYP16365*, *CYP16671* and *CYP18835* has correlation with azadirachtin accumulation in different tissues and their developmental stages.

### Characterization of members of CYP gene family putatively associated with azadirachtin biosynthesis

Full-length cDNA of CYP450s, which showed correlation (*AiCYP16671*, *AiCYP16365* and *AiCYP18835*) for accumulation of azadirachtin were isolated from cDNAs prepared from fruit tissue using gene-specific primers (Supplementary Table S4). The full-length ORF of CYP16671, CYP16365 and CYP18835 encoded polypeptides of 504, 508 and 466 amino acids residues respectively. The theoretical isoelectric point and molecular weight of CYP16671 were 5.01 and 55 kDa. The isoelectric point of CYP16365 and CYP18835 was 5.02 and the molecular weight was 55 kDa and 51 kDa respectively. The polypeptide chain of CYP16671 consisted of two transmembrane helices; one located between A_4_(P) and A_23_(L) and the other located between A_302_(M) and A_324_(M). The polypeptide chains of CYP16365 and CYP18835 consisted of one transmembrane helix each; which was located between A_5_(I) and A_22_(L) in CYP16365 and in case of CYP18835 between A_7_(V) and A_24_(L). The signal peptide cleavage site was predicted to be present in between A_29_ and A_30_ in CYP16671 and between A_25_ and A_26_ in CYP16365; however, no signal peptide cleavage site was observed in case of CYP18835. Secondary structure prediction indicated that all these members of the CYP450s consisted of alpha helices, extended strands, beta turns and random coils. CYP16671 consisted of alpha helix (40.91%), extended strand (17.79%), beta turn (9.09%) and random coil (32.21%); CYP16365 consisted of alpha helix (46.46%), extended strand (13.58%), beta turn (9.25%) and random coil (30.71%); CYP18835 consisted of alpha helix (48.07%), extended strand (16.31%), beta turn (7.30%) and random coil (28.33%).

The conserved domains present across all these CYPs, included P450 domain, CypX (domain important in Cytochrome P450 for secondary metabolite biosynthesis, transport and catabolism, defense mechanism), P450-cyclo_AA_1 domain, Carotene-beta-ring hydroxylase domain and P450-derived glycosyltransferase activator domain. There was variation in three CYPs, where CYP16671 consisted of PL03234 domain (Cytochrome P450 83B1); CYP16365 consisted of PLN02183 domain (ferulate-5-hydroxylase) and CYP18835 consisted of PLN02186 domain (abscisic acid-8′-hydroxylase). These three CYPs showed 70–85% similarity with characterized CYP450s of other plants of different families.

**Figure 6:**
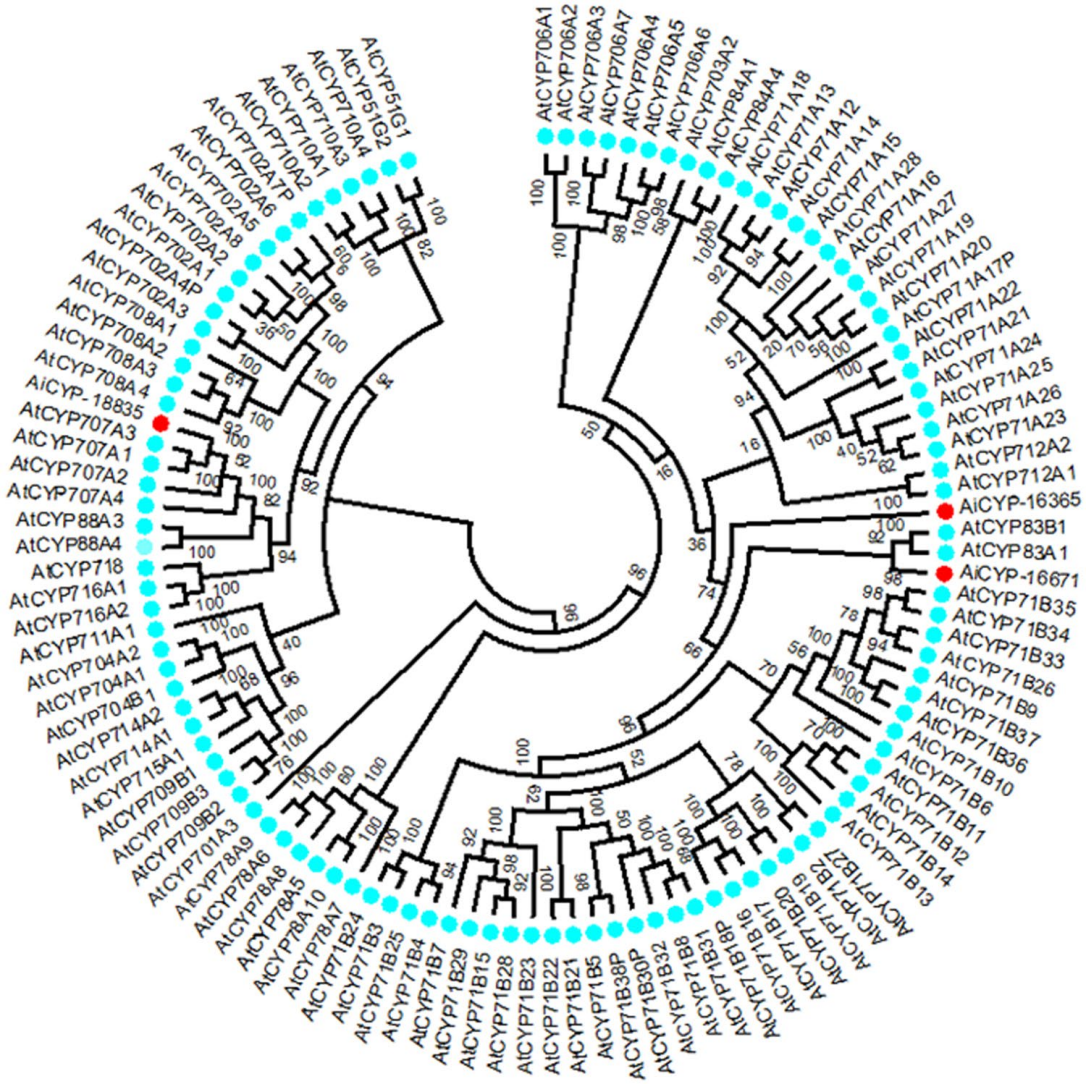
Phylogenetic analysis of genes encoding members of CYP450 family putatively involved in triterpenoid biosynthesis. Phylogenetic tree was constructed for CYP16671, CYP16365 and CYP18835 and members of CYP450 families present in *Arabidopsis thaliana* by neighbor-joining method using MEGA 5 software. CYP450s of *A*. *indica* are indicated by red dots and CYP450s of *A*. *thaliana* are indicated by cyan colored dots.

A phylogenetic tree was constructed by neighbor-joining method using MEGA 5 software to gain more information regarding evolutionary relationship of identified CYPs against Cytochrome P450s of *Arabidopsis thaliana* belonging to different families (Fig. 6). Plant P450s are generally classified into A- and non-A type clades, which are further classified into families and then subfamilies. CYP16671, CYP16365 and CYP18835 clustered with CYP83B, CYP71B and CYP707A families respectively, suggesting close evolutionary relationships with the members of these groups.

### Molecular Docking analysis

**Figure 7:**
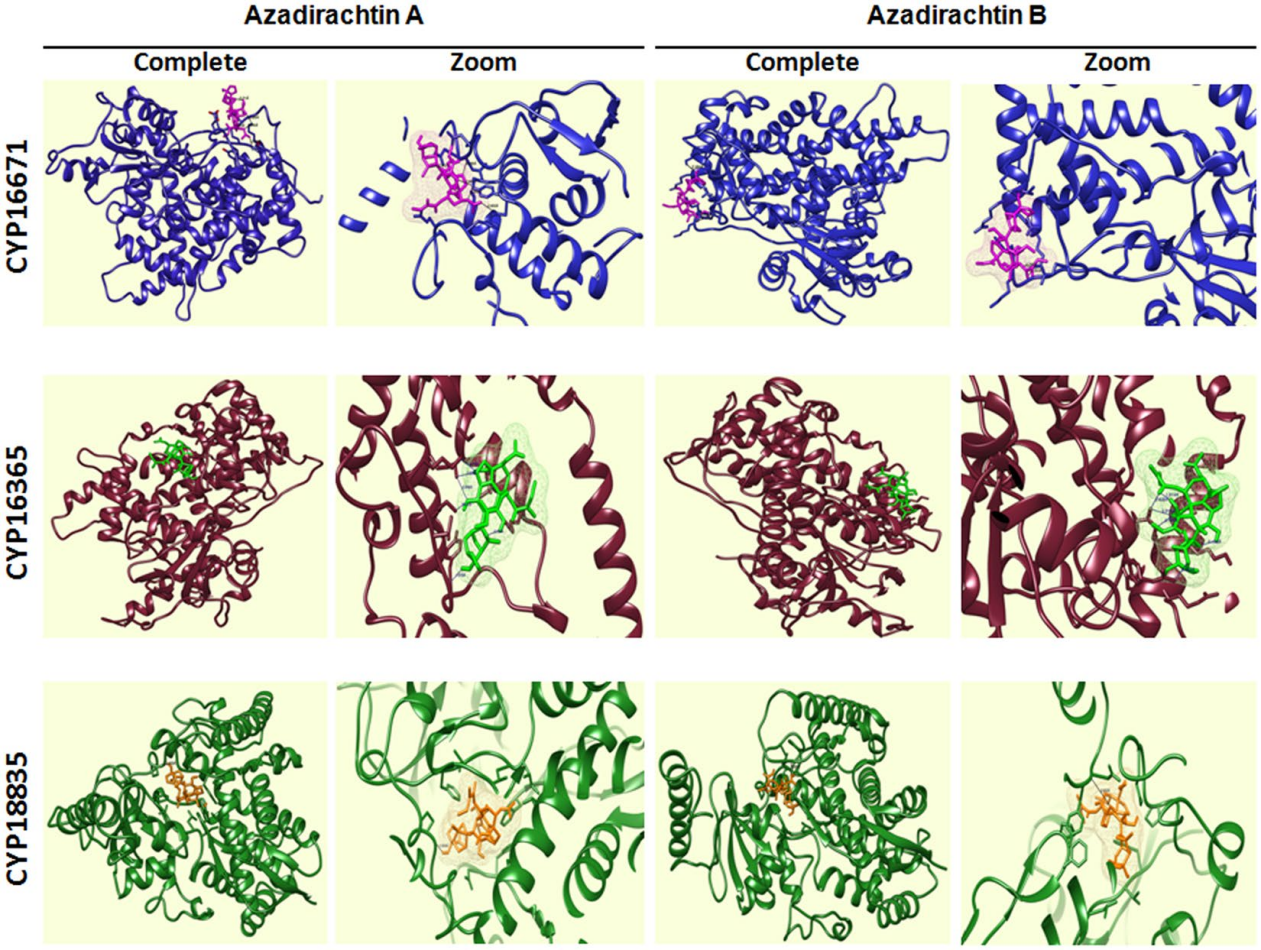
Molecular docking analysis of CYP450 putatively involved in triterpenoid biosynthesis. Interaction of complete protein and docked regions with Azadirachtin A and Azadirachtin B are shown in the figure. Three CYP450s are represented in different colors. Complete block representing the whole protein ligand complex, whereas the zoom blocks are showing the interacting orientation of functional moieties of Azadirachtin A and Azadirachtin B within the cavity of protein.

Molecular docking of three CYP proteins (CYP16671, CYP16365 and CYP18835) was performed with eleven triterpenoids (ligands) (Fig. 7 and Supplementary Figs S1–S3). Analysis revealed that binding energy was lowest in case of CYP16671 docked with nimbanal and epoxyazadiradione forming one and two hydrogen bonds respectively. However, azadirachtin A and B formed stable complexes with CYP16671 with four and three hydrogen bonds with −6.01 and −5.75 kcal/mol of binding energies (Supplementary Table S5). The docking analysis for CYP16365 revealed that, among all the ligands, binding energy was lowest for salannin and nimbinene with −10.14 and −10.05 kcal/mol forming one hydrogen bond for each molecule. However, azadirachtin A and B formed stable complexes with four and five hydrogen bonds respectively, indicating that the conformation of azadirachtin A and B is best suitable for CYP16365 when number of hydrogen bonds formed between protein and ligand is set as criteria (Supplementary Table S6). The hydrogen bonds between azadirachtin A and CYP16365 are formed at ASN432, LYS362, LYS429 and LYS358 with ligand moiety at different positions (Supplementary Table S6) with varying bond length (Fig. 7). The energy minimization graph of ligands shows that all the ligands minimized with CYP16365 and CYP16671 have almost similar energy stabilization pattern (Supplementary Fig. S4). Docking of CYP18835 with triterpenoids showed interaction through only one hydrogen bond with binding energy of −10.43 and −9.85 kcal/mol for azadirachtin A and azadirachtin B (Fig. 7 and Supplementary Table S7).

## Discussion

Neem tree is known as natural pharmacy and contains tetranortriterpenoids as the biologically active principles synthesized via isoprenoid pathway. For getting insights into the biosynthesis of important metabolites, genomic information regarding this medicinal plant has been established^15, 16, 18^. Analysis of these genomic resources has provided limited information about the biosynthesis of intermediate steps and requires developing detailed information about biosynthesis of bioactive molecules, correlation between expression of genes and metabolite content needs to be established. In past, such correlation between metabolite content and transcriptome data has provided information regarding biosynthetic pathways for medicinally important molecules^11, 19, 20^. In this study, tissue-specific synthesis of azadirachtin has been correlated with expression of genes to establish biosynthetic pathway. We carried out transcriptome sequencing of leaf (ML) and fruit (FS3) tissues, as azadirachtin specifically accumulated in the fruit, with highest accumulation in FS3 rather than leaf tissue (Fig. 1).

Establishment of transcriptome datasets and assembly yielded 8,49,781 and 7,07,392 high quality reads for ML and FS3 respectively. In *Withania somnifera*, transcriptome assembly of leaf and root yielded 6,75,691 and 7,31,352 high quality reads^11^ and pyrosequencing of glandular trichomes of *Artemesia annua* yielded 4,06,044 reads^21^. In past, analysis of transcriptome datasets generated information about various intermediate steps involved in synthesis of bioactive molecules from plants with high medicinal importance^22, 23^. Classification of unigenes present in leaf and fruit datasets by GO assignments revealed that a large number of unigenes was designated to ‘other metabolite processes’ suggesting that established data can be useful in the identification of genes participating in secondary metabolite biosynthesis of *A*. *indica* (Fig. 2). KEGG analysis led to the functional classification and pathway assignment, which showed that among all the pathways, the cluster of ‘limonene and pinene degradation’ represented the largest group. Differential gene expression analysis suggested differential expression of a large number of genes between leaf and fruit tissues (Fig. 3). Expression analysis of genes of MVA and MEP pathways by qRT-PCR showed variation in relative expression in leaf and different developmental stages of fruit tissues of *A*. *indica* (Fig. 4). Genes of MVA pathway (*AiHMGR2* and *AiHMGS*) showed higher transcript levels in ripened stage of fruit (FS4) as compared to leaf, which may explain the increased participation of MVA pathway (rather than MEP pathway) in the formation of triterpenoid backbone for the production of metabolites in fruit tissue revealing the importance of enzymes in the formation of triterpenoids of Neem. Out of two genes encoding HMGR, we have already shown a correlation of expression with azadirachtin content and higher activity of *AiHMGR2* as compared to *AiHMGR1* ^24^.

Tirucallol is believed to be the precursor of triterpenoids present in *A*. *indica* ^12^. Though genomic resources have been developed, genes involved in secondary modifications taking place in triterpenoid backbone and intermediate steps for the synthesis of azadirachtin and related triterpenoids of Neem are uncharacterized. We used transcriptome datasets developed in this study, to identify CYP450s which might play an important role in secondary modifications and synthesis of azadirachtin. Formation of triterpenoids of Neem involves oxidation, hydroxylation and epoxidation reactions and azadirachtin, which is the active principle of plant, accumulated in fruit tissue. Differential expression analysis led to the identification of a set of CYP450, which might be involved in azadirachtin as these were several folds up-regulated in fruit as compared to leaf tissue. Validation of their expression by qRT-PCR showed correlation of three CYP450 with azadirachtin accumulation in fruit tissue (Fig. 1).

Phylogenetic analysis of AiCYP16671 showed similarity to *SAUR2* gene encoding CYP83B1, which catalyzes the metabolism of aromatic oximes in glucosinolate biosynthesis and probably required in response to pathogen and functions in auxin homeostasis in *Arabidopsis* ^25^. AiCYP16365 shows similarity with enzymes belonging to CYP71B family which is known to participate in several pathways including ascorbate and alderate metabolism; limonene and pinene degradation; and coumarine and phenylpropanoid biosynthesis. *AtPAD3* gene encoding CYP71B15, from this family, catalyzes final step in the biosynthesis of camalexin, which is the main phytoalexin in *Arabidopsis thaliana* ^26^. In addition, AiCYP18835, which correlated with the azadirachtin accumulation showed similarity with CYP707A1 gene encoding Abscisic acid-8′hydroxylase^27^.

The participation of AiCYPs (CYP16671, CYP16365 and CYP18835) in triterpenoid biosynthesis pathway was analyzed by docking approaches to study the enzyme-substrate interactions. Favorable interaction between the protein and ligand was observed by negative binding energies, as tested for eleven triterpenoids. The highest number of H-bonds was formed with azadirachtin A and azadirachtin B with AiCYP16671 and AiCYP16365. The number of bonds and the distance between enzyme and substrate showed that azadirachtin A and azadirachtin B fit into the active site cavity of CYP16671 and CYP16365. Molecular docking and dynamics simulation are useful in studying the interaction of protein and substrates. In *W*. *somnifera*, WsSGTL1 and WsSGTL2 have been shown to interact with sterols and withanolides predicting the possible role of these two important glycosyl transferases in glycosylation mechanism of sterol glycosides in *W*. *somnifera* ^28^. Computational modeling along with functional analysis provided in-depth information about the unusual catalytic behavior of AsCYP51H10 of *Avena strigosa* and its participation in avenacin biosynthesis^29^.

### Conclusions and future perspective

We have studied tissue specific distribution of triterpenoids present in *A*. *indica*, as this tree is considered as natural pharmacy consisting of signature metabolites, tetranortriterpenoids. Transcriptome datasets established from leaf and fruit tissues generated information on the molecular basis for identification of genes involved in biochemical reactions of the MVA and MEP pathways. The expression analysis of these genes revealed the increased participation of genes of MVA pathway as compared to MEP pathway in the biosynthesis of fruit-specific triterpenoids of *A*. *indica*. The expression analysis of genes encoding CYP450s indicated the involvement of three AiCYPs in azadirachtin biosynthesis since the expression of *AiCYP16671*, *AiCYP16365* and *AiCYP18835* strongly correlated with azadirachtin accumulation in fruit tissue. Molecular docking approaches revealed strong binding of azadirachtin A and azadirachtin B to AiCYP16671 and AiCYP16365 with maximum number of hydrogen bonds giving a strong prediction about the involvement of these two CYPs in azadirachtin biosynthesis. Further characterization of these CYPs is required to confirm their participation in uncharacterized steps of azadirachtin biosynthesis pathway.

## Methods

### Plant material

Different developmental stages of fruit (FS1, FS2, FS3, FS4) and leaf of *A*. *indica* ^24^ were collected from five-year old tree growing in the botanical garden of CSIR-National Botanical Research Institute, Lucknow, India (11°24′N/79°44′E). After collection and washing with sterile water, plant tissues were sampled in liquid nitrogen and stored in −70 °C until use. For phytochemical profiling through HPLC, plant parts were air-dried and powdered.

### Phytochemical analysis

Fine powder of plant parts (air-dried) was employed for the extraction and phytochemical analysis according to Bhambhani *et al*.^24^. Standard azadirachtin and salannin/nimbin were purchased from Sigma-Aldrich (Sigma-Aldrich, USA) and Trifolio-M GmbH, (Lahnau, Germany) respectively. The average content of the molecules was calculated for at least three independent samples of each tissue and expressed in terms of percentage of dry weight of the tissue using replicates.

### Preparation of cDNA libraries, sequencing and annotation

High quality total RNA extracted using Spectrum Plant Total RNA Kit (Sigma-Aldrich, USA) from frozen tissues (leaf and FS3) was utilized for cDNA library preparation. The procedure of double-stranded (ds) cDNA library preparation and sequencing was according to Gupta *et al*.^19^ using 454-GS FLX sequencing platform (454 Life Sciences, Roche, USA) using GS FLX Titanium Kit. Quality-filtering algorithm for filtering of raw read sequences from leaf and fruit libraries and annotation of contigs and singletons from individual leaf and fruit libraries as well as combined assembly was carried out according to Gupta *et al*.^19^. Annotation was carried out against Arabidopsis protein database at The Arabidopsis Information Resource (TAIR; http://www.arabidopsis.org;Tair10) and the NCBI non-redundant protein (NR) database (http://www.ncbi.nlm.nih.gov).

### GO annotation and pathways assignment

Functional assignment of each of the unigene generated from combined assembly was carried out using GO annotation in online TAIR Database (http://www.arabidopsis.org/). The TAIR IDs of all the unigenes were utilized for the classification into the groups of Cellular Component, Molecular Function and Biological Process. To assign into metabolic networks, mapping of unigenes from combined assembly into various metabolic pathways was performed according to the Kyoto Encyclopedia of Genes and Genomes (KEGG). All the unique sequences were assigned with an enzyme commission (EC) number on the basis of BLASTx search of protein databases, having an E-value cut-off of 10^−5^.

### Digital differential gene expression analysis

Gene families involved in the biosynthesis of terpenoid backbone as well as CYP450 were annotated and used for the digital gene expression profiling between leaf and fruit tissues. To statistically determine the differential gene expression the R statistics^30^ was applied and R ≥ 8 were considered to be highly significant. To calculate the threshold R value, 1000 datasets for each library were generated according to the random Poisson distribution as described previously^30^.

### Identification of members of gene families involved in biosynthesis of triterpenoids of *A*. *indica*

Contigs related to genes involved in MVA and MEP pathways as well as for the triterponoid backbone biosynthesis were identified by searching gene or enzyme name for each enzymatic step in annotation results. Full-length sequence of the identified genes encoding enzymes catalyzing different steps of MVA and MEP pathways (upto farnesyl diphosphate synthase) and those involved in secondary conversion (members of CYP450 gene family) were obtained using assembled data of *A*. *indica* leaf and fruit transcriptomes (SRP008022 and SRP008023)^15^ generated through illumina sequencing platform. Digital gene expression among leaf and fruit tissues of *A*. *indica* was studied by using log_2_ converted values, as representation of reads to form each contig associated with biosynthesis of terpenoid backbone, and heat maps were constructed by using MeV (version 4.8.1).

### Quantitative gene expression analysis

Quantitative expression of genes was analyzed using RT-PCR System and Fast SYBR Green PCR Master Mix (ABI 7500, Applied Biosystems, USA). For each primer set a control reaction having no template was also included. Data from qRT-PCR amplification was analyzed using comparative Ct (2^−ΔΔCt^) method^31^ to calculate fold change in expression. For statistical analyses, experiments were carried out in triplicates using three biological as well as three technical replicates. Gene specific oligonucleotides used for qRT-PCR analysis are provided in Supplementary Table S4.

### Isolation and molecular cloning of full-length *AiCYP450s*

Total RNA was isolated from fruit using Spectrum Plant Total RNA Kit (Sigma-Aldrich, USA) followed by treatment with RNase-free DNase I 146 (Ambion, USA) according to the manufacturer’s instructions. A first strand cDNA was prepared using PolyA enriched RNA, 500 ng oligo dT primer and RNase H^−^ Revert Aid MulV Reverse transcriptase (Fermentas, Life Sciences, USA). Full-length open reading frame of CYP16671, CYP16365 and CYP18835 was amplified from cDNA of fruit by using primers covering complete open reading frame (Supplementary Table S4). The fragments were cloned into pTZ57R/T vector followed by sequencing. Primers were designed based on whole genome and transcriptome sequence of *A*. *indica* downloaded from the database (NCBI, http://www.ncbi.nlm.nih.gov)^15, 32^.

### Bioinformatic analysis of *AiCYP450s* and encoding protein

The full-length nucleotide sequence of identified genes encoding CYP450s were subjected to BLAST analysis using nucleotide database available at NCBI (http://www.ncbi.nlm.nih.gov/) for sequence similarity searches. The open reading frame (ORF) of identified CYP450s was predicted by using Translate tool (http://www.expasy.ch/tools/dna.html/). The deduced nucleotide and polypeptide sequence alignments were performed using ClustalW programme (http://www.ebi.ac.uk/clustalW/). Analysis of trans-membrane domains along the polypeptide stretch was carried out by TMHMM 2.0 server (http://www.cbs.dtu.dk/services/TMHMM-2.0/). The secondary structure of peptides of AiCYP450s was analyzed by SOPMA (https://npsa-prabi.ibcp.fr/cgi-bin/npsa_automat.pl?page=/NPSA/npsa_sopma.html). The isoelectric point (pI) and molecular weight of the deduced CYP450 proteins were predicted by the software pI/Mw Tool at http://www.expasy.org. To gain insight into the evolutionary relationship of CYP450s, AiCYP450s were set as a query to search the database of NCBI by using BLASTp searches and the amino acid sequences of characterized CYPs from *Arabidopsis thaliana* were retrieved. The phylogenetic tree was constructed by neighbor-joining method using MEGA5 software^33^. The reliability of the tree was measured by bootstrap analysis with 1000 replicates.

### Molecular modelling and docking for enzyme-substrate analysis

Modelling of AiCYPs (CYP16671, CYP16365 and CYP18835) was performed by using Phyre2 web portal using fold recognition method^34^. Molecular Dynamics Simulation (MD) was performed using GROMOS56 force field in GROMACS for structure refinement of models of AiCYPs (Supplementary Figure S4). Docking analysis was performed to predict the interactions of different triterpenoids (ligand) as substrate for the identified CYP450 proteins (CYP16671, CYP16365 and CYP18835) using AutoDock 4.0 (http://autodock.scripps.edu). For covering acting domains present in CYP450 protein, grid spacing was maintained at 0.375 Å. Genetic algorithm (GA) was applied as searching parameter with 10 GA runs and population size was set to 150, energy evaluations was set to maximum 25,00,000 with considering the maximum number of generations to 27,000. The most favorable docking conditions were in the form of lowest binding energy conformations with H-bonds in cluster. UCSF-Chimera molecule viewer tool was used for better analysis of interaction in protein-ligand complexes obtained from AUTODOCK4^28^ software.

## Electronic supplementary material

Supplementary Information
