# Supplementary material for: Transcriptome and metabolite analyses in Azadirachta indica: identification of genes involved in biosynthesis of bioactive triterpenoids
Source: Sci Rep. 2017 Jul 11;7:5043. doi: 10.1038/s41598-017-05291-3 (PMC5505991; doi:10.1038/s41598-017-05291-3)
Supplement: Supplementary file 1 — Supplementary Information [file 41598_2017_5291_MOESM1_ESM.pdf]

**Supporting information**

# Transcriptome and metabolite analyses in *Azadirachta indica:* identification of genes involved in biosynthesis of bioactive triterpenoids

Sweta Bhambhani[1,2], Deepika Lakhwani[1,2], Parul Gupta[1,#], Ashutosh Pandey[1,$], Yogeshwar Vikram Dhar[1,2], Sumit Kumar Bag[1,2], Mehar Hasan Asif[1,2,*], Prabodh Kumar Trivedi[1, 2,*]

[1]CSIR-National Botanical Research Institute, Council of Scientific and Industrial Research (CSIR-NBRI), Rana Pratap Marg, Lucknow-226 001, INDIA

[2]Academy of Scientific and Innovative Research (AcSIR), Anusandhan Bhawan, 2 Rafi Marg, New Delhi-110 001, India

# Present address (PG): Botany & Plant Pathology, Oregon State University, Corvallis-97331, USA

[$]Present address (AP): Faculty of Biology, University of Bielefeld, Universitätsstraße 25, 33615 Bielefeld, Germany

[*]Author for correspondence:

PKT: prabodht@nbri.res.in; prabodht@hotmail.com

MHA: mh.asif@nbri.res.in

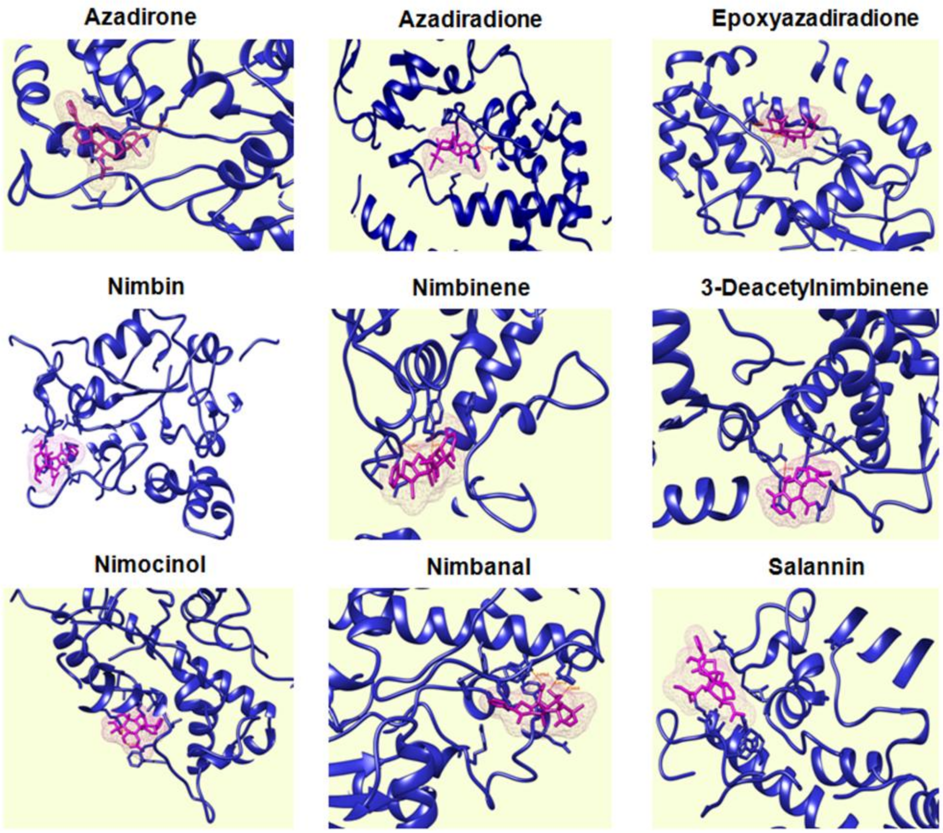


**Supplementary Figure S1.** Molecular docking of protein encoded by *AiCYP16671* with different ligands (Zoom view).

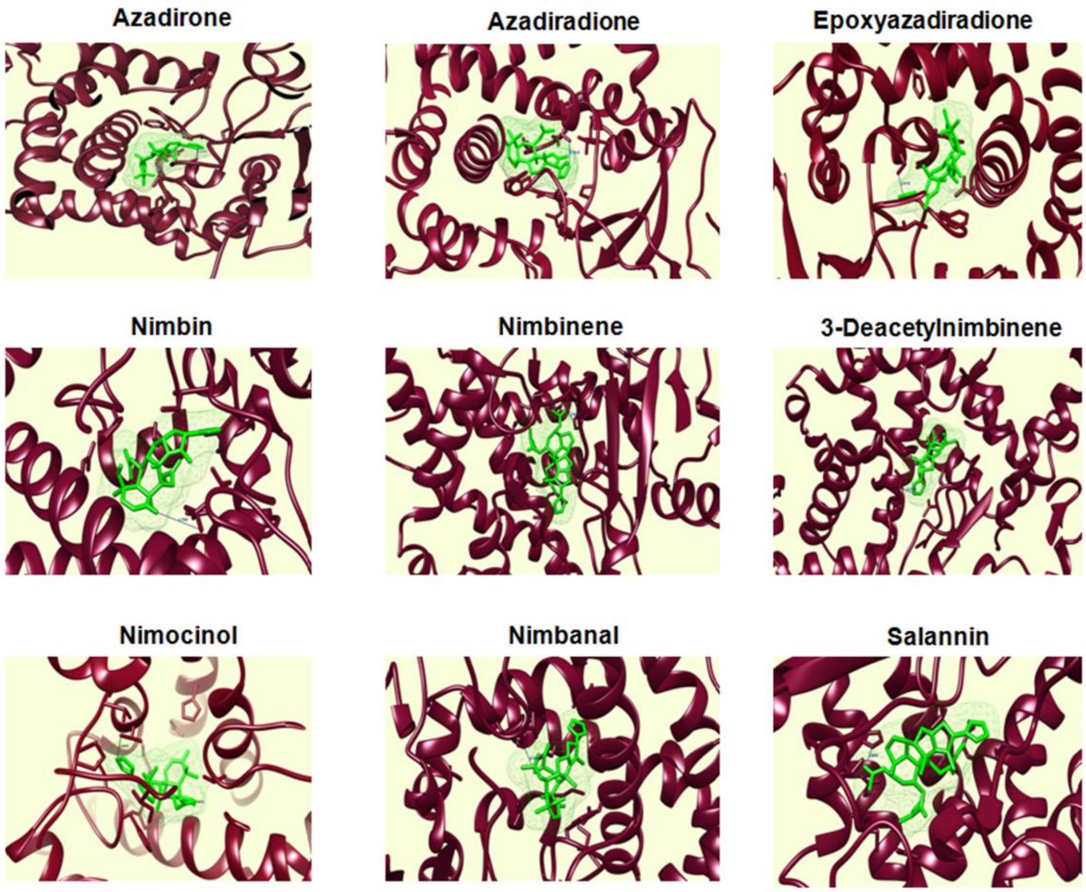


**Supplementary Figure S2.** Molecular docking of protein encoded by *AiCYP16365* with different ligands (Zoom view).

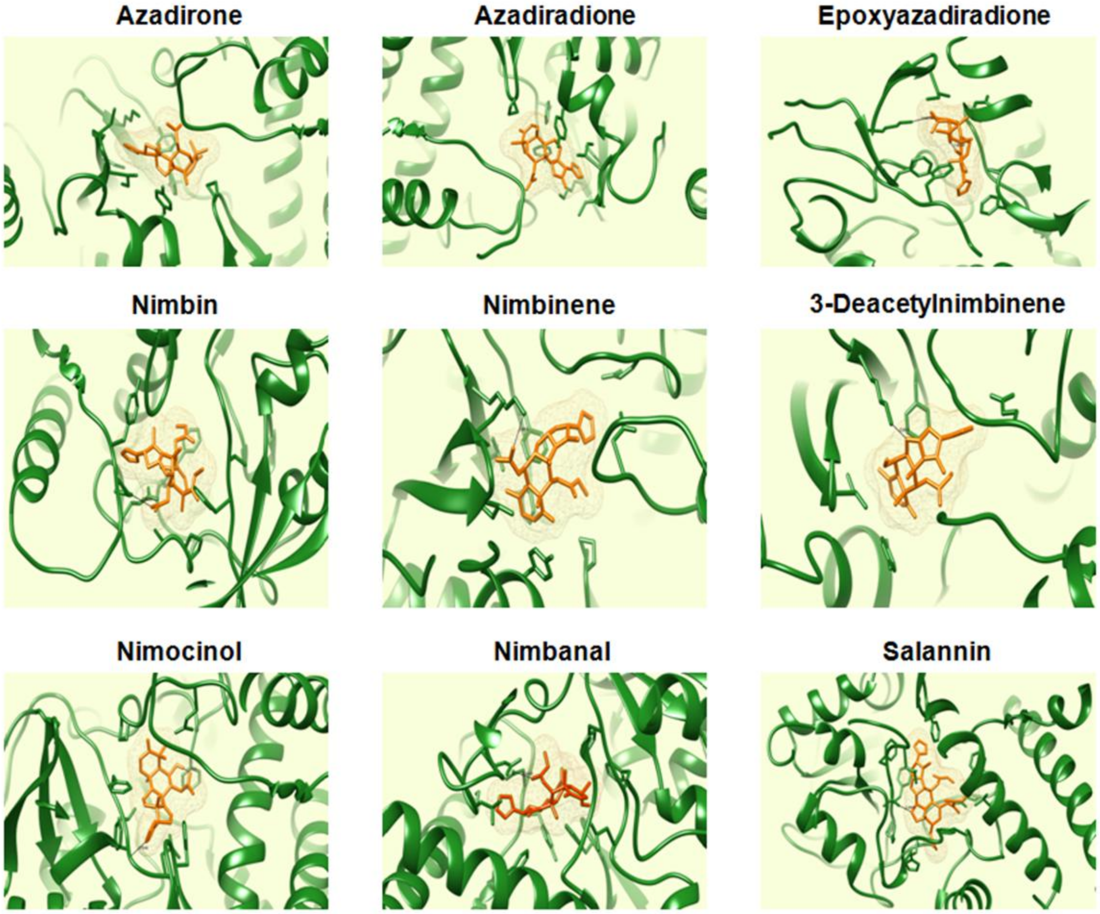


**Supplementary Figure S3.** Molecular docking of protein encoded by *AiCYP18835* with different ligands (Zoom view).

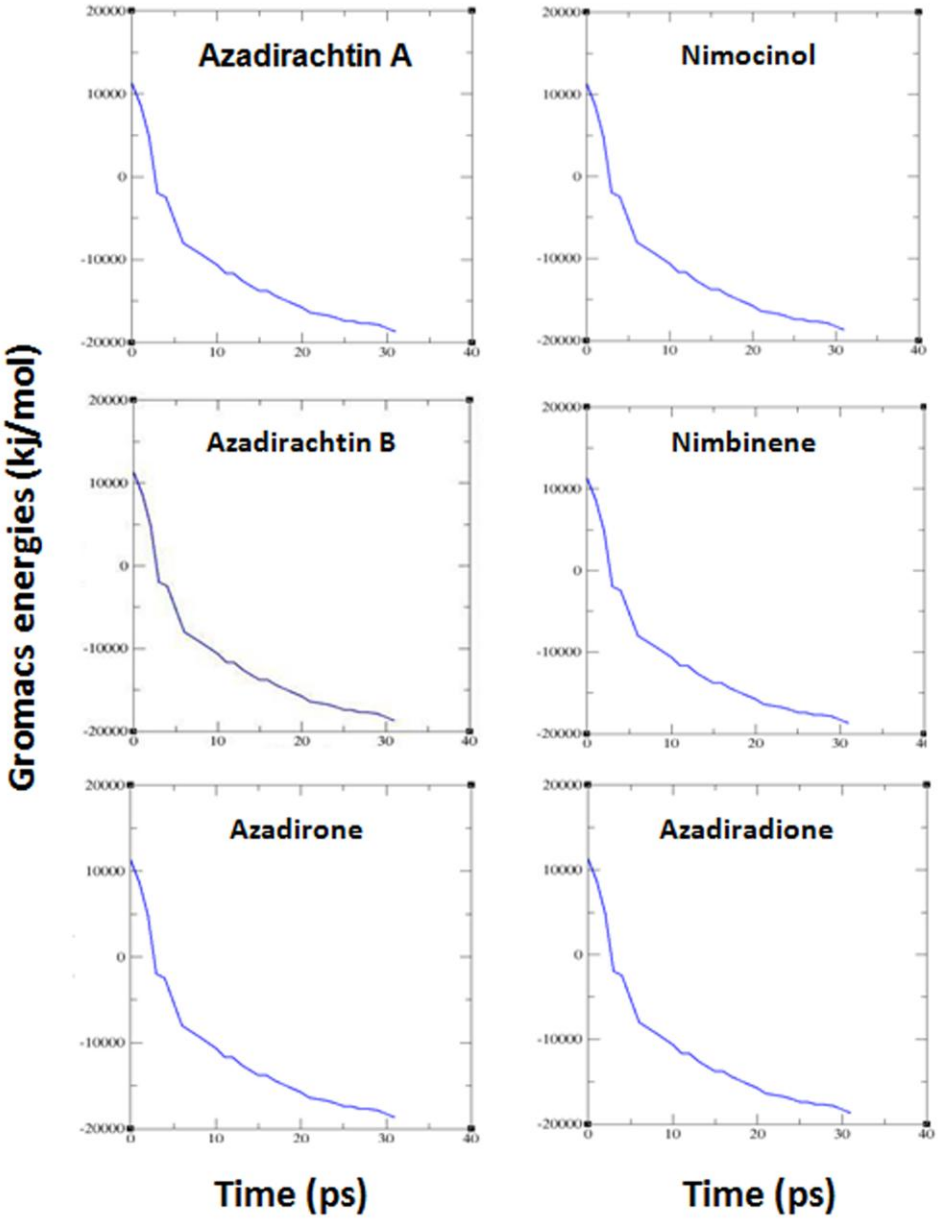


**Supplementary Figure S4.** Energy minimization of AiCYP16671 with different triterpenoids.

**Table S1.** Details of analysis of 454 sequencing and assembly for leaf and fruit of *Azadirachta indica*

| | **Fruit** | **Leaf** |
|---|---|---|
| HQ reads | 707392(219432334 bp) | 849781(273414110 bp) |
| Average HQ read length | 310.9 bp | 322.2 bp |
| Reads assembled as contigs | 563212 | 697725 |
| Number of contigs | 14566(10008059 bp) | 18172(13309111bp) |
| Average length of contigs | 687.1 bp | 732.4 bp |
| Range of contigs length | 100-3771 bp | 100-4042 bp |
| Contigs above 200bp | 13483 | 16838 |
| Average GC% | 41% | 41.1% |
| Number of Singletons | 46310(12921686 bp) | 48163(14271393bp) |
| Average length of singletons | 279 | 296.3 |
| Range of singleon lengths | 50-601 bp | 50-577 bp |
| Singletons above 200 bp | 35013 | 39664 |
| Average GC% | 39.90% | 39.8% |

**Table S2.** Annotation details of fruit and leaf transcriptomes using various databases.

| | **Fruit** | **Leaf** |
|---|---|---|
| Total transcripts | 60876 | 66335 |
| Annotated by AGIprot | 30385 | 34898 |
| Annotated by NR | 33960 | 38977 |
| EST Scan passed | 35532 | 40353 |
| Total no. of gene families by Pfam | 20110 | 23265 |

**Table S3.** Contigs encoding different gene families annotated against different databases.

| Gene Families | Fruit | Leaf |
|---|---|---|
| Cytochrome P450 | 419 (149 contigs, 270 Singletons) | 352 (170 contigs, 182 Singletons) |
| Methyl transferases | 23 (10 contigs, 13 Singletons) | 28 (13 contigs, 15 Singletons) |
| Glycosyl transferase | 63 (25 contigs, 28 Singletons) | 57 (27 contigs, 28 Singletons) |

**Table S4.** List of primers used in the present study.

| S.No. | Primer name | Forward Sequence (5' to 3') | Reverse Sequence (3' to 5') |
|---|---|---|---|
| 1. | *AiHMGSRT* | TTGGCTCCAGGAACATACTATCTTAC | CTCTTAGTGACCATTTGAGAGTGAAC |
| 2. | *AiHMGR1RT* | GTTCTGTTCTGGCAGCAGAACTCTC | CCACCTCACCATCTATGATGCAGATG |
| 3. | *AiHMGR2RT* | GGAGAGTTATCTCTCATGTCTGCTC | CATCTTTATGGACTTCCTGTACCAC |
| 4. | *AiDXSRT* | CTGTTGATGGTCACAACATAGATGAC | GGTACTTGTCTGCAGCCTTCTCAGC |
| 5. | *AiDXRRT* | GCTTTGAATTTGCTATCTCCAGCTG | AATGAAGTTCCACCATCCTTCCTCTTC |
| 6. | *AiFPPSRT* | TATCAGAATATGAGTGATCTGCATTC | GGCACATTGTAGTCCAGCATACGTCT |
| 7. | *AiSQSRT* | AAATCTTACATAATCCAACGTGAGC | TAAGTTACAATAAGGTAATCACTAG |
| 8. | *AiCYP16365Full1* | ATCCAGATTATAAACTTCATTC | CAAAACGAGACTGAATTGACCAC |
| 9. | *AiCYP16365Full2* | AACTTCATTCAATTGACATGGAG | AAGTAGAGATACCACGGCGTCC |
| 10. | *AiCYP16671Full1* | GTACTCGTTGCAGTTGCCCTTATC | TAACATACAAACTTCAGCCAAAC |
| 11. | *AiCYP16671Full2* | GTTGCCCTTATCATAATAATAAGAATG | CTTAATTTACGGTCCACGGGCC |
| 12. | *AiCYP18835Full1* | GCACCACCACTACTACTACTGCCT | TTGCTATCTGTTCTGCTGTG |
| 13. | *AiCYP18835Full2* | CTGCCTCTACTTCCACTTCTCACG | CTGTGGATACGCGAAAAGTGCT |
| 14. | *AiCYP16671RT* | TTGAAGCTCTACCTGGTATGGCTG | CAAACGAGGATGGCTGTGTAAGTG |
| 15. | *AiCYP19051RT* | GAATTAATACCTTTGGCTCAGGAAG | CATGTCAACCGGTTCATCACCTAC |
| 16. | *AiCYP16365RT* | TGTGCATCATTCCTATTCCTTATC | AACGAGACTGAATTGACCACATTG |
| 17. | *AiCYP09513RT* | TCTTTGTATGCATTGTTGCTCCAG | TTGCTGTAATGGAGTCAACTGTAG |
| 18. | *AiCYP18835RT* | ACCCAATACGTTCATGCCATTTG | TGTGGTCAGATGATGAAGAAGAAC |
| 19. | *AiCYP15747RT* | TTCGTCACTCTCTGCCTCTGCTTC | TTCGGCAACCCAGTCATGGAAGCG |
| 20. | *AiCYP16469RT* | TTATATGGCTGGTGGGCTGAAG | CAAATCCAATCAAGTGGTTGGCTC |
| 21. | *M13* | TGTAAAACGACGGCCAG | CAGGAAACAGCTATGACC |

**Table S5.** Details of molecular docking of AiCYP16671 protein with ligands.

| Ligands | Binding Energy | Ligand Efficiency | Inhibition constant | Total internal energy | H-bonds Number | H-bonds | Ligand Moiety |
|---|---|---|---|---|---|---|---|
| Azadirone | -8.01 | -0.25 | 1.36 | -1.07 | 1 | PHE417:O | OAC |
| Nimocinol | -8.01 | -0.25 | 1.36 | -1.07 | 1 | PHE417:O | OAC |
| Azadiradione | -7.63 | -0.23 | 2.55 | -1.24 | 1 | LYS325:HZ3 | OAB |
| Epoxyazadiradione | -8.04 | -0.24 | 1.27 | -1.15 | 2 | LYS363,ASN43 | O1,OAB |
| Nimbin | -6.79 | -0.17 | 10.54 | -3 | 0 | 0 | 0 |
| 6,Deacetylnimbin | -8.01 | -0.25 | 1.35 | -1.35 | 1 | TYR322:OH | HAB |
| Nimbanal | -9.11 | -0.25 | 209.36 | -10.99 | 3 | LYS325:HZ3,LEU480:HN,ASN326:HD22 | OAH,OAD,OAB |
| Salannin | -6.56 | -0.15 | 15.62 | -3.44 | 0 | 0 | 0 |
| Nimbinene | -7.82 | -0.22 | 1.85 | -2.21 | 2 | LYS325:HZ1,LEU480:HN | OAD,OAA |
| AzadirachtinA | -6.01 | -0.12 | 41.3 | -5.46 | 4 | LYS325:HZ1, LEU480:HN,ASN326:HD22,LYS325:HZ3 | OAA,HAH |
| AzadirachtinB | -5.75 | -0.11 | 60.77 | -5.45 | 3 | LYS332:HZ3,LYS325HZ2,TYR357:HH | OAP,OAG,OAG |

**Table S6.** Details of molecular docking of AiCYP16365 protein with ligands.

| Ligands | Binding Energy | Ligand Efficiency | Inhibition constant | Total internal energy | H-bonds Number | H-bonds | Ligand Moiety |
|---|---|---|---|---|---|---|---|
| Azadirone | -9.55 | -0.3 | 99.29 | -1.21 | 1 | THR111:HG1 | OAC |
| Nimocinol | -9.97 | -0.3 | 49.43 | -2.18 | 1 | THR111:HG1 | OAC |
| Azadiradione | -9.68 | -0.29 | 80.71 | -1.27 | 1 | THR111:HG1 | OAD |
| Epoxyazadiradione | -9.53 | -0.28 | 103.38 | -1.13 | 1 | THR111:HG1 | OAD |
| Nimbin | -6.54 | -0.17 | 16.05 | -2.88 | 1 | LYS362:HZ1 | OAH |
| 6-Deacetylnimbin | -9.09 | -0.28 | 216.93 | -1.78 | 1 | THR310:HN | OAE |
| Nimbanal | -8.66 | -0.23 | 445.49 | -2.53 | 1 | HIS242:HE2 | OAF |
| Salannin | -10.14 | -0.24 | 37.07 | -2.28 | 1 | HIS242:HE2 | OAI |
| Nimbinene | -10.05 | -0.29 | 43.22 | -2.26 | 1 | THR310:HG1 | OAF |
| AzadirachtinA | -5.9 | -0.12 | 47.85 | -5.04 | 4 | ASN432:O,LYS362:HZ1,LYS429:HZ1,LYS358 HZ1 | HAG,OAE,OAC |
| AzadirachtinB | -5.55 | -0.11 | 85.68 | -5.2 | 5 | LYS96:HZ1,LYS362:HZ1,GLY441:HN,TYR435:O,LYS358:HZ2 | OAK,OAG,OAP,HAE |

**Table S7.** Details of molecular docking of AiCYP18835 protein with ligands.

| **Ligands** | **Binding Energy** | **Ligand Efficiency** | **Inhibition constant** | **Total internal energy** | **H-bonds Number** | **H-bonds** | **Ligand Moiety** |
|---|---|---|---|---|---|---|---|
| Azadirone | -10.95 | -0.34 | 9.39 | -1.17 | 0 | 0 | 0 |
| Nimocinol | -10.81 | -0.33 | 11.84 | -1.95 | 1 | GLU110:OE2 | H1 |
| Azadiradione | -11.03 | -0.33 | 8.23 | -1.06 | 0 | 0 | 0 |
| Epoxyazadiradione | -10.07 | -0.3 | 41.79 | -1.13 | 1 | LYS369:NZ | OAC |
| Nimbin | -9.48 | -0.24 | 112.33 | -3.26 | 1 | ASN207:ND2 | OAI |
| 6,Deacetylnimbin | -9.26 | -0.29 | 163.84 | -1.78 | 1 | LYS369:NZ | OAA |
| Nimbanal | -9.93 | -0.27 | 52.89 | -2.64 | 1 | ASN207:ND2 | OAF |
| Salannin | -10.14 | -0.24 | 36.91 | -2.88 | 1 | THR349:OG1 | OAA |
| Nimbinene | -10.48 | -0.3 | 20.95 | -2.24 | 2 | ASN207:ND2,LYS369:NZ | OAD,OAG |
| AzadirachtinA | -10.43 | -0.2 | 22.61 | -5.5 | 1 | PRO106:O | HAG |
| AzadirachtinB | -9.85 | -0.19 | 60.7 | -5.55 | 1 | THR349:OG1 | HAG |
